# Exploring the Idea of Human Reproduction in Space: A Potential Area for Future Research

**DOI:** 10.7759/cureus.73712

**Published:** 2024-11-14

**Authors:** Priyanka Sharma, Sunita Malik, Avir Sarkar

**Affiliations:** 1 Obstetrics and Gynecology, Employees' State Insurance Corporation (ESIC) Medical College and Hospital, Faridabad, Faridabad, IND

**Keywords:** cosmic radiation, microgravity, outer space, space gynecology, space travel

## Abstract

As space travel evolves from brief missions to longer expeditions, and with the rise of space tourism, there is increasing interest in understanding how space travel affects human reproductive physiology and the feasibility of procreation in space. Space travel presents various potential hazards to reproductive health such as exposure to ionizing radiation, exposure to changes in gravity, psychological stress, and disruptions to the endocrine and urogenital systems, as well as the circadian rhythm. This article explores how cosmic radiation and microgravity impact both female and male gametogenesis, embryogenesis, and reproductive physiology.

## Introduction and background

“Life, uh, finds a way….”

Jeff Goldblum’s famous words from the movie Jurassic Park take on new significance as we stand on the brink of a thrilling but challenging frontier: space tourism. The evolution of species is neither linear nor predictable, and humanity has faced and overcome countless challenges our environment presents. Now, as we prepare to explore and perhaps even colonize the skies, our fragile bodies are presented with yet another formidable frontier: the vast, uninhabitable expanse of space.

Since the first manned mission in 1961, more than 600 individuals have ventured into space, with only 24 traveling beyond low Earth orbit [1]. Of these, only 11% have been women [1], and there is limited knowledge of how space travel affects reproductive health. As space agencies like National Aeronautics and Space Administration (NASA) transition from short missions to longer expeditions and space tourism grows, attention must be directed toward understanding how space travel influences reproductive physiology and the possibilities and challenges of procreation in space. Space Medicine, a growing specialty, focuses on studying the effects of space travel on human biology.

## Review

Space stressors

Space travel poses numerous hazards to both male and female reproductive health, including exposure to ionizing radiation, variations in gravitational forces, psychological stress, and disruptions to the endocrine and urogenital systems, along with circadian rhythm disturbances. While some of these stressors exist during long-haul flights, the human body, while Earth-bound, is unfamiliar with two critical influences: microgravity and cosmic radiation.

The Earth’s magnetic field and atmospheric shield protect us from high-energy cosmic radiation, which consists of photons, protons, helium nuclei, and high-charge, high-energy particles [2]. In space, travelers face exposure to both galactic cosmic radiation and solar particle events, depending on mission destination, vehicle type, duration, and solar conditions. Exposures may range from 54 to 108 mSv in low Earth orbit to up to 1070 mSv for interplanetary travel [3]. Ionizing radiation can cause DNA damage and generate oxidative stress, which is associated with several reproductive disorders. The gonads, highly sensitive to radiation, are particularly vulnerable, and high-dose radiation exposure can lead to temporary infertility in men.

Humans are adapted to thrive under Earth’s gravity (1g). However, space travelers experience microgravity aboard space stations, zero gravity in space, and hyper-gravity during spacecraft takeoff and re-entry. With limited opportunities for human studies, researchers rely on cell cultures, tissue cultures, and animal models to simulate space conditions [4]. These studies show that both real and simulated microgravity influence cellular processes such as differentiation, proliferation, migration, survival, and apoptosis [5]. Microgravity has also been shown to impact cell adhesion, signaling, and pro-survival pathways [6]. Intriguingly, the 3D multicellular spheroids formed under microgravity conditions could serve as metastasis models, aiding cancer research and treatment [7].

Effects of space travel on female reproductive health

Female astronauts have traditionally used oral contraceptives during space missions to suppress ovulation, avoid personal hygiene and waste disposal challenges, reduce menstrual symptoms, and prevent bone loss [8]. Given the demands of space travel, many female astronauts choose to suppress menstruation for extended periods, ranging from 9 to 11 years. The use of combined hormonal pills, subdermal implants, and the levonorgestrel-releasing intrauterine system is considered suitable for these circumstances [9]. Female astronauts often delay pregnancy until after space travel, with the mean age for first space travel being 36 years. Consequently, many opt for assisted reproductive technology (ART) due to advanced maternal age [8]. There appears to be no significant difference in ART outcomes between female astronauts and those who have not travelled to space. It was reported that the mean age of female astronauts at the time of pregnancy was 40 years, and the mean age for spontaneous abortion was 41 years [8].

However, extrapolating data from this small cohort of highly screened, healthy women to the general population may introduce bias. None of the female astronauts have travelled beyond low Earth orbit, and no human conception or childbirth has yet occurred in space. Animal studies, however, provide some insight into the effects of space travel on reproduction. For example, microgravity has been shown to reduce estrous cycling and impair pregnancy outcomes in rats [10,11]. In simulated microgravity environments, ovarian follicles and oocytes experience structural and functional disruptions, suggesting that the organization of the meiotic spindle may be gravity dependent [12,13].

In studies where mice were exposed to simulated microgravity, the rate of oocyte maturation was significantly reduced (8.94% versus 73.0%), suggesting a gravity-dependent role in meiotic spindle organization, though the structure and function of microtubules remained unaffected [13]. Preimplantation embryonic development occurred in true space flight conditions, but blastocyst development rates and quality were severely impaired. The first study of pregnancy during space flight involved rats aboard unmanned biosatellites. Out of five rats, two became pregnant but showed signs of resorption. While the rats that gave birth had litter sizes similar to those of the control group, fetal weight, placental weight, and skeletal development were delayed. Four of five rats delivered live offspring, while one delivered a stillborn. Labor was prolonged for all rats, and the mortality rate for the litter was higher during the first postnatal week. However, the surviving female offspring reached adulthood and reproduced successfully [10].

Data from pregnant mice flown on the COSMOS 1514, NASA-NIH R1, and NASA-NIH R2 missions showed that mid-to-late gestational exposure to space flight did not disrupt fetal development or parturition, though some variability in pregnancy duration, neonatal weight, and litter size was observed [14-16]. Both male and female offspring from these litters were fertile and successfully reproduced. Space flight, however, resulted in decreased luteinizing hormone and follicle-stimulating hormone levels [14]. A more recent study involving adult female mice aboard the International Space Station for 37 days showed no differences in estrous cycling or ovarian estrogen concentrations, though progesterone levels were lower in space-exposed mice compared to controls [17].

The DNA damage sustained by embryos during space flight was comparable to low-dose radiation exposure on Earth, suggesting that radiation, rather than gravitational changes, was the primary cause of damage [18]. Microgravity caused delays in blastocyst formation and reduced the number of tropho-ectodermal cells in the embryo. Embryos exposed to microgravity had a live birth rate less than half that of embryos developed under normal gravity conditions [19]. Luteal cells cultured in microgravity showed increased DNA damage, decreased progesterone secretion, increased apoptosis, and fewer active mitochondria [20]. Oyama and Platt in a study found, under hyper-gravity simulation conditions, a decreasing pregnancy rate in rats exposed to increased gravity, with no offspring surviving extended centrifugation [21].

While no direct links between space travel and cancer risk have been established, the relationship between high-dose radiation exposure and ovarian cancer is well known. NASA lists “Space Radiation Carcinogenesis” as a top research priority, highlighting the need to establish new radiation exposure standards for longer duration space missions.

Effect of space travel on male reproductive health

Male astronauts experience decreases in total and free testosterone levels after 12-13 days in space [22]. While spermatogonial stem cells are more resistant to radiation than actively dividing spermatogonia, radiation exposure can still lead to temporary decreases in sperm count and degeneration of seminiferous tubules, which are presumed reversible upon returning to Earth [23,24]. Studies of simulated microgravity effects on spermatogenesis have shown reduced epididymal sperm count and seminiferous tubule degeneration. Space radiation is known to cause oxidative damage to testicular germ cells, activate p53, and induce apoptosis in germ cells [25].

To date, no instances of intercourse in space have been reported, and the biomechanics of sexual intimacy in altered gravity remain largely unexplored. This area of study poses a wealth of fascinating questions yet to be addressed.

## Conclusions

In conclusion, space travel alters fundamental physiological processes, and humans will need to adapt to these influences to enable successful procreation and childbirth in space. Space stressors affect everything from the cytoskeleton of single cells to mesenchymal migration, vestibular adaptation, and skeletal remodeling, all of which could impact human embryonic development. Although little is known about the effects of space travel on reproductive health, the success of recent missions demonstrates that space biology is an emerging and promising field of study. The notion of inhabiting space has captivated human imagination for centuries. Beyond biological challenges, space exploration raises numerous ethical and moral questions, such as reproductive rights, intimacy in space, and issues of equity and social justice. Now is the time to forge ahead into this uncharted territory and establish the foundations for human life in space.

## References

[1] (2023). Astronauts & cosmonauts. https://www.worldspaceflight.com/.

[2] Dietze G, Bartlett DT, Cool DA (2013). ICRP Publication 123: Assessment of radiation exposure of astronauts in space. Ann ICRP.

[3] Cucinotta FA, Kim MH, Willingham V, George KA (2008). Physical and biological organ dosimetry analysis for international space station astronauts. Radiat Res.

[4] Tou J, Ronca A, Grindeland R, Wade C (2002). Models to study gravitational biology of mammalian reproduction. Biol Reprod.

[5] Grimm D, Wehland M, Corydon TJ (2020). The effects of microgravity on differentiation and cell growth in stem cells and cancer stem cells. Stem Cells Transl Med.

[6] Monti N, Masiello MG, Proietti S (2021). Survival pathways are differently affected by microgravity in normal and cancerous breast cells. Int J Mol Sci.

[7] Grimm D, Schulz H, Krüger M (2022). The fight against cancer by microgravity: the multicellular spheroid as a metastasis model. Int J Mol Sci.

[8] Jennings RT, Baker ES (2000). Gynecological and reproductive issues for women in space: a review. Obstet Gynecol Surv.

[9] Jain V, Wotring VE (2016). Medically induced amenorrhea in female astronauts. NPJ Microgravity.

[10] Serova LV (1989). Effect of weightlessness on the reproductive system of mammals. (Article in Russian). Kosm Biol Aviakosm Med.

[11] Ronca AE, Baker ES, Bavendam TG, Beck KD, Miller VM, Tash JS, Jenkins M (2014). Effects of sex and gender on adaptations to space: reproductive health. J Womens Health (Larchmt).

[12] Cheng K, Feng X, Yang C (2023). Simulated microgravity reduces quality of ovarian follicles and oocytes by disrupting communications of follicle cells. NPJ Microgravity.

[13] Wu C, Guo X, Wang F (2011). Simulated microgravity compromises mouse oocyte maturation by disrupting meiotic spindle organization and inducing cytoplasmic blebbing. PLoS One.

[14] Burden HW, Zary J, Lawrence IE, Jonnalagadda P, Davis M, Hodson CA (1997). Effects of space flight on ovarian-hypophyseal function in postpartum rats. J Reprod Fertil.

[15] Burden HW, Poole MC, Zary J, Jeansonne B, Alberts JR (1998). The effects of space flight during gestation on rat uterine smooth muscle. J Gravit Physiol.

[16] Ronca AE, Alberts JR (2000). Physiology of a microgravity environment selected contribution: effects of spaceflight during pregnancy on labor and birth at 1 G. J Appl Physiol (1985).

[17] Hong X, Ratri A, Choi SY, Tash JS, Ronca AE, Alwood JS, Christenson LK (2021). Effects of spaceflight aboard the International Space Station on mouse estrous cycle and ovarian gene expression. NPJ Microgravity.

[18] Ronca AE, Moyer EL, Talyansky Y (2019). Behavior of mice aboard the International Space Station. Sci Rep.

[19] Wakayama S, Kawahara Y, Li C, Yamagata K, Yuge L, Wakayama T (2009). Detrimental effects of microgravity on mouse preimplantation development in vitro. PLoS One.

[20] Yang H, Bhat GK, Sridaran R (2002). Clinostat rotation induces apoptosis in luteal cells of the pregnant rat. Biol Reprod.

[21] Oyama J, Platt WT (1967). Reproduction and growth of mice and rats under conditions of simulated increased gravity. Am J Physiol.

[22] Smith SM, Heer M, Wang Z, Huntoon CL, Zwart SR (2012). Long-duration space flight and bed rest effects on testosterone and other steroids. J Clin Endocrinol Metab.

[23] Sapp WJ, Philpott DE, Williams CS, Williams JW, Kato K, Miquel JM, Serova L (1992). Comparative study of spermatogonial survival after x-ray exposure, high LET (HZE) irradiation or spaceflight. Adv Space Res.

[24] Masini MA, Albi E, Barmo C (2012). The impact of long-term exposure to space environment on adult mammalian organisms: a study on mouse thyroid and testis. PLoS One.

[25] Li H, He Y, Yan J, Zhao Q, Di C, Zhang H (2016). Comparative proteomics reveals the underlying toxicological mechanism of low sperm motility induced by iron ion radiation in mice. Reprod Toxicol.

